# Risk factors of healthcare-associated infection among healthcare workers in intensive care units: A multicenter cross-sectional study

**DOI:** 10.1371/journal.pone.0314796

**Published:** 2024-12-17

**Authors:** Salah Alshagrawi, Norah Alhodaithy

**Affiliations:** 1 Department of Public Health, College of Health Sciences, Saudi Electronic University, Riyadh, Saudi Arabia; 2 King Fahad Medical City, Riyadh, Saudi Arabia; Freelance Consultant, Myanmar, MYANMAR

## Abstract

**Background:**

Healthcare-associated infections (HAIs) are a major global health threat, leading to higher morbidity and mortality, longer hospital stays, and increased healthcare expenses. Intensive care units (ICUs) present a particularly high risk of developing HAIs. This study aims to examine the risk factors of HAIs among healthcare workers (HCWs) in the ICUs of selected public hospitals.

**Methods:**

We employed a cross-sectional design using an online survey. Respondents were randomly selected from seven large public hospitals located in different areas of Riyadh, the capital city of Saudi Arabia. Data collection was conducted between November 1st to 15th, 2023. Logistic regression analysis was employed to examine previous exposure to HAIs as the response variable and selected predictors.

**Results:**

A total of 600 HCWs participated in the study (response rate 88.2%). Among the study HCWs, 75.1% were female, with nurses making up 50% of the sample. Of the respondents, 78% had at least a year’s experience, 71% had applied infection prevention and control (IPC) training from the infection control department, and 93% reported they had good knowledge about infection control. The level of knowledge of IPC (OR = 0.9, *p* < 0.05) and applied IPC training (0.1, *p* < 0.001) were significantly associated with a lower risk of HAIs. Additionally, a higher risk of HAIs was associated with HCWs years of clinical experience (*p* < 0.001).

**Conclusion:**

Overall, the findings indicated that HCWs who have poor knowledge of IPC, who reported no previous IPC applied training, and who have more years of clinical work experience have a greater risk of HAIs. Thus, legislators and Health officials should prioritize the prevention of infections linked to healthcare, paying particular attention to tailored and applied IPC initiatives.

## Introduction

Healthcare-associated infections (HAIs) are a major global health threat, leading to higher morbidity and death, as well as longer hospital stays and healthcare expenses [1,2]. Moreover, HAIs raise the incidence of resistance to antibiotics [3]. Some infections, such as pneumonia, bloodstream infection, and urinary tract infection, can develop into serious life-threatening diseases [4]. Healthcare facilities bear the burden of high treatment expenses and prolonged hospital stays to address the incidence of such illnesses [5]. The most prevalent disease among those linked to HAI is pneumonia, which makes up between 30% and 50% of all infections [6]; bloodstream infections are common, with a frequency of 4% to 20% [7]; Urinary tract infections can occur in 3–7% of cases [8]. These infectious consequences may pose an additional strain for the individual suffering from the illness, resulting in a rise in the burden of disease and the creation of an underlying sepsis disorder [9].

In Intensive care units (ICUs), there is a particularly high risk of developing infections associated with HAI. Patients in critical care units are particularly vulnerable to infections due to the invasive nature of their therapies and weakened immune systems. Despite admitting fewer patients than other departments in the Healthcare facilities, the ICUs have between 2 and 2.5 greater frequency of HAIs [10] because patients hospitalized in the ICUs are more vulnerable because they may suffer from serious injuries, loss of consciousness, or insufficient immune response to infections [11]. It is estimated that 20% to 50% of illnesses in ICUs are attributed to HAIs [12]. The percentage of HAIs varied by hospital unit; infections among ICU patients were found to be 19.5% more common than those in other units, which were 5.2% in other units. As a result, it is estimated that 56.5% of patients in ICU have high and essential usage of antibiotics due to preventable infections [13]. According to estimates from the World Health Organization (WHO), 30% of patients in critical care units who have one or more instances of infections caused by healthcare suffer adverse outcomes [14].

Notably, between 20% and 30% of these infections result from preventable causes [15]. Thus, preventing infections linked to healthcare is essential for raising treatment standards, providing the potential to save lives, and minimizing expenses [16]. The occurrence of HAI can be attributed to the ICU setting [17], medical interventions implemented for the patient’s treatment, and primarily, the patient’s overall health. Patients in ICUs receive treatment for serious illnesses with underlying medical conditions and concomitant illnesses may make them more susceptible to infections produced by HAIs [17]. Intubation tubes, urethral catheters, and central vascular lines are examples of intrusive devices that are required to be used and maintained over time due to the necessity of performing several diagnostic and therapeutic procedures on patients. This might lead to the elimination of the body’s natural defense mechanism against infections. [18]. As a result, it is critical to determine the causes and risk factors of infections, as well as understand the processes by which infections spread among healthcare professionals and patients in the ICU.

The purpose of this study is to examine the risk factors of Healthcare-Associated Infection among healthcare workers in the ICUs of several public hospitals in Riyadh, Saudi Arabia. Recognizing the risk factors of HAIs would reduce the duration of hospital stays, cut death rates, minimize expenditures on pharmaceuticals and supplies, and alleviate the illness burden for patients, particularly in cases related to sepsis.

## Methods

### Study design and setting

We employed a cross-sectional design with a web-based anonymous HCWs Data collection was conducted between November 1st to 15th, 2023 in the ICUs of seven public hospitals in Saudi Arabia, located in busy areas in different parts of the capital city of Riyadh. The hospitals had an average capacity of 800 beds with a 1:4 average nurse-to-patient ratio.

### Study participants

A total of 680 HCWs, health professionals, health associate professionals, personal care workers in health services, and support personnel, who work in the ICU received the invitation to participate in the online survey, and 600 of these individuals responded (response rate = 88.2%). The method of probabilistic sampling was employed for the random selection of respondents, and it was determined based on the hospital infection control center’s data in the register of ICU hospital employees. A minimal sample size of 580 was determined by utilizing a 5% margin of error, a 95% confidence level, a 50% response rate, and an 80% prior estimate level [18]. Probabilistic sampling assures the sample accurately reflects the population, enables researchers to assess the degree of uncertainty in the results, and facilitates the generalization of findings to the population. All HCWs, health professionals, health associate professionals, and support personnel, who work in the ICU were eligible to participate in the study.

### Data collection

The recruited respondents received email invitations with a wealth of information about the study’s goals, the anticipated time it would take to complete the survey, the researcher’s contact information, privacy and confidentiality assurances, and the requirement to sign a written informed permission form. A self-reporting, anonymous questionnaire was developed based on the literature [19]. The questionnaire included general respondent characteristics such as respondent age, sex, specialty, years of clinical work experience, self-efficacy, knowledge of infection prevention and control (IPC), previous applied IPC training, time pressure and workload, and availability of IPC resources.

### Primary outcome variable

The primary outcome variable was the proportion of HCWs who had previous HAI incidents. The variable has been developed to examine the proportion of HCWs who had experienced an incident of HAIs in the time while working in their current hospital in the ICU. The incidence of HAIs was measured by asking the respondents whether they had been involved in any type of HAI incident while working in the ICUs in their current hospital. The item has only two options: Yes or No.

### Potential determinants and risk factors

Based on the literature, we adopted several risk factors such as years of experience in healthcare, healthcare profession specialty, the accessibility, and availability of essential materials and resources to prevent HAIs, knowledge of infection prevention and control (IPC), self-efficacy, previous IPC applied training, time pressure, and workload, and availability of IPC resources. Table 1 presents the items and reliability scores for self-efficacy in performing HAIs measures (3 items), time pressure and workload (6 items), and availability of IPC resources (6 items), variables which were measured based on a Likert scale with five points, ranging from "strongly disagree" to "strongly agree". Time constraints and workload were defined as the failure to comply with HAI protocols due to distractions, insufficient time, and engagement with other responsibilities. The availability of IPC resources was defined by possessing the essential mandate and being used to ensure effective HAI measures. Each construct was measured by adding the points of the construct’s items.

**Table 1:** Items for study measures with descriptive statistics.

Construct	Measurement Item	λ	α	CR	AVE	Range	Mean	SD
Self-efficacy	Generally, I am confident in my ability to perform hand hygiene.	0.83	0.84	0.81	0.62	3–15	9.56	3.01
	Despite the complexity of my job, I believe I can protect myself and others from HAIs	0.86						
	Despite the complexity of my job, I have the skills to protect myself and others from HAIs	0.88						
Time pressure and workload	I sometimes forget to do hand hygiene.	0.91	0.86	0.85	0.65	5–25	17.92	6.92
	The needs of my patients during emergencies take priority over doing hand hygiene.	0.90						
	I sometimes forget to do hand hygiene because I get distracted by other things.	0.82						
	I am often too busy to perform hand hygiene.	0.84						
	It is difficult for me to go to hand hygiene training due to time pressure.	0.82						
	I do not have time to do hand hygiene as often as I am supposed to.	0.88						
Availability of infection control Resources	Alcohol-based hand rub is located in convenient areas.	0.83	0.93	0.91	0.75	6–30	24.63	5.98
	Dedicated sinks for healthcare workers to do hand hygiene are located in convenient areas.	0.86						
	Dedicated sinks for healthcare workers to do hand hygiene always have liquid soap and paper towels readily available.	0.84						
	Alcohol-based hand rub dispensers are always full.	0.88						
	Alcohol-based hand rub is always located at point-of-care.	0.93						
	Moisturizer is located in convenient areas.	0.91						

Note. λ = Standardized factor loadingl; α = Cronbach’s alpha reliability coefficient; CR = Composite reliability; AVE = Average variance extracted.

### Statistical analysis

The participant characteristics were analyzed using descriptive statistics such as means, standard deviations, and frequencies. Inferential statistics were used to explore the relationships between variables and assess the relationship between HAI incidents and their potential determinants and risk factors. We specifically conducted a logistic regression analysis with previous exposure to HAIs as the response variable and sex, years of experience in healthcare, healthcare profession specialty, knowledge of IPC, self-efficacy, previous IPC applied training, time pressure and workload, and availability of infection control resources as explanatory factors. The logistic regression model’s goodness-of-fit was evaluated using a likelihood ratio test to examine the impact of the risk factors on HAIs. All statistical tests employed in this study were two-tailed, and a p-value less than 0.05 was considered statistically significant. The quantitative data from the survey questionnaire was analyzed using the Statistical Package for the Social Sciences (SPSS).

### Ethical considerations

The study was approved by the King Fahad Medical City Institutional Review Board under number 1R800010471 and Federal Wide Assurance number FWA00018774. After recruiting participants, the research objectives were clarified to them, and prior to starting the interview, informed written consent was secured before any audio recording commenced. All participants were made aware of the confidentiality of their data and their right to join or withdraw from the study at any time.

## Results

Table 2 presents the respondents’ demographic characteristics. 75.1% of the study’s HCWs were female, with nurses making up 50% of the sample. Of the respondents, 78% had at least a year’s experience, 71% had attended IPC applied training from the infection control department, and 93% indicated having a good level. Table 2 shows the statistical characteristics of the three constructs: Self-efficacy, available resources, time pressure, and workload. Reliability indicators explain the degree to which all items in a questionnaire assess the same idea or construct. For instance, self-efficacy showed good internal reliability (α = 0.8), Composite Reliability (CR = 0.8); Average Variance Extracted (AVE = 0.6). Time pressure and workload variables have similar acceptable indicators, internal reliability (α = 0.9), Composite Reliability (CR = 0.9); Average Variance Extracted (AVE = 0.7), Respondents’ Self-efficacy in adherence to IPC measures ranged between 3 to 15 with an average of 9.7 (SD = 3). The overall score for the available resource constructs varied from 6 to 30, with an average of 24.63 (SD = 5.9). The time pressure and workload construct varied from 5 to 25, with an average of 17.92 (SD = 6.9).

**Table 2:** Respondents’ characteristics (N = 600).

Characteristic	Description	N (%)
Healthcare Profession Category	Laboratory Specialist	20 (3.3)
	Nurse	300 (50)
	Pharmacist	12 (2.0)
	Physical therapist	34 (5.7)
	Physician	44 (7.3)
	Radiology Therapist	26 (4.3)
	Respiratory Therapist	56 (9.3)
	Other	108 (18)
Sex	Female	446 (74.3)
	Male	154 (25.7)
Years of Experience in Healthcare	Less than 1 year	50 (8.3)
	1–5 years	158 (26.3)
	6–10 years	158 (26.3)
	More than 10 years	234 (39)
How would you rate your knowledge of infection control	Good	558 (93)
	Poor	42 (7)
Have you had any previous infection control clinical training from the infection control department	No	174 (29)
	Yes	426 (71)
have been involved in any type of HAI incident while working in the ICU in their current hospital	No	358 (59.7)
	Yes	242 (40.3)

Of the respondents, 40% indicated they or their patients had experienced a prior HAI incidence while working in their current position. The results showed a significant chi-squared statistic of χ^2^(8) = 20.8, *p* < .001. This suggests that a model containing risk factors as predictors fits the data significantly better than one without them. Furthermore, the model’s R^2^ value of 0.3 signifies a notable enhancement compared to the null model, reflecting the proportion of variance in the outcome variable that is collectively accounted for by the explanatory variables. In other words, the model demonstrates that it accounts for nearly 32% of the variability between healthcare workers who experienced HAI incidence and those who did not.

Table 3 shows the results of the logistic regression model. The findings showed a significant odds ratio (OR) of 0.7 for the respondent’s knowledge about IPC (95% CI; 0.4, 0.9), this indicates that HCWs who have proper knowledge of IPC have a 30% decrease in the risk of HAIs compared to HCWs with poor knowledge of IPC. Another significant determinant was having applied IPC training from the infection control department (ICD). An OR of 0.1 for respondents who had applied IPC training, (95% CI; 0.1, 0.2) indicates that HCWs with no training have an 88% increased risk of HAI compared to HCWs with prior applied hands-on training in IPC. Additionally, the respondent’s experience has shown a significant impact. HCWs who have 10 or more years of experience have shown greater odds compared to other experience categories (OR = 0.3; 95% CI; 0.1, 0.4). The remaining risk factors did not show any significant findings.

**Table 3:** Findings of the Logistic regression of HAI incident and potential risk factors.

Variable		B	SE	*P*-value	Exp (B)	95% Confidence Interval for Exp (B)	
						Lower Bound	Upper Bound
Sex (Male)		0.20	0.24	0.40	1.22	0.76	1.97
Healthcare Profession Category (Nurse)	Laboratory Specialist	0.83	0.55	0.12	2.30	0.79	6.78
	Pharmacist	-1.21	0.84	0.15	0.30	0.06	1.54
	Physical therapist	0.71	0.43	0.10	2.04	0.88	4.76
	Physician	-0.22	0.51	0.67	0.81	0.30	2.19
	Radiology Therapist	0.34	0.33	0.30	1.41	0.74	2.67
	Respiratory Therapist	0.71	0.43	0.10	2.04	0.88	4.76
Years of Experience in Healthcare (More than 10 years)	Less than 1 year	-0.32	0.37	< 0.001*	0.72	0.35	1.50
	1–5 years	-0.72	0.26	0.01*	0.48	0.29	0.81
	6–10 years	-0.47	0.26	0.04 *	0.63	0.38	0.98
How would rate your infection control knowledge (Good)		- 0.10	0.30	0.04*	0.78	0.43	0.91
Have you had any previous infection control training (Yes)		-2.11	0.32	< 0.001*	0.121	0.06	0.23
Perceived self-efficacy construct		-0.12	0.08	0.11	0.88	0.76	1.03
Availability of Resources construct		-0.02	0.02	0.31	0.98	0.95	1.02
Perceived time pressure and workload construct		0.03	0.03	0.33	1.03	0.97	1.10

## Discussion

Our findings showed that almost two-thirds of the respondents had received applied training in IPC, and half of the respondents had previously encountered HAIs. Respondents perceived self-efficacy was about average and most of them reported adequate available resources to practice IPC. Receiving applied training, the respondents’ clinical experience, and appropriate knowledge about IPC were all significantly associated with HAIs.

We found that HCWs who have good knowledge of IPC have a lowered risk of HAIs compared to HCWs with poor knowledge of IPC [20]. Insufficient knowledge about IPC regulations contributes to heightened HAIs and decreased IPC compliance increasing the likelihood of diseases among HCWs and patients and increasing the financial burden on the healthcare system [21,22]. Poor compliance results from a lack of information regarding the appropriateness, efficacy, and use of IPC measures [23–25]. Hand hygiene, applying personal protective equipment (PPE), vaccination against transmissible infections, patient infection examination, clinical instrument disinfection, and managing medical waste are all important factors that HCWs should be informed about [26]. HCWs’ inadequate understanding of IPC has been associated with a decline in healthcare service outcomes [27–29]. Education is the foundation of reducing HAIs by improving IPC procedures [30,31]. Despite the provided education, a lack of knowledge of IPC procedures has been frequently demonstrated [32]. Thus, it is suggested that HCWs have a comprehensive understanding of IPC through the participation of educational courses and ongoing education programs [33,34]. Additionally, campaigns or initiatives that are coordinated nationally and have been demonstrated to effectively promote IPC, guarantee the use of methods and recommendations, and positively impact HCWs’ knowledge of IPC to reduce HAIs [35].

In our study, HCWs who reported no previous applied training showed an increased risk of HAI compared to HCWs with prior training in infection control. Applied IPC training, such as simulation-based teaching, is an effective educational method that produces greater and longer-lasting outcomes and is linked to lower HAI and improved hand hygiene compliance [36–38]. Mostly, IPC training tends to be confined to classroom settings, the primary method of providing IPC training to HCWs [39]. Such methods might not be sufficient to increase HCW’s skills and competencies. Additionally, a universal strategy will not be appropriate for all organizations. For instance, it has been identified that in low-resource situations, the effectiveness of IPC training is usually diminished [40,41]. More IPC compliance and improved performance are more likely to result from targeted and customized IPC training programs [42,43]. Thus, Monitoring, assessing, and gathering feedback from HCWs would substantially enhance the quality, development, and delivery of IPC training [44,45]. While most ICP programs offer training, they do not mandate that HCWs demonstrate a particular level of competence or skills in IPC correct practice [46,47]. Many countries demand IPC education and training through hospital accreditation regulations and requirements, but there is no obligation to evaluate compliance [48]. A thorough strategy for IPC practice and performance assessment is required.

Our findings showed that HCWs who have 10 or more years of clinical work experience have a greater risk of HAIs compared to those with fewer years of experience categories. This could be due to the lack of interest among senior HCWs in engaging in IPC training. Other studies have shown inconsistent findings. Several studies revealed that HCWs’ clinical work experience greatly influenced their knowledge and attitudes about IPC adherence, which contributed to a lower risk of HAIs [49,50] In other research, however, career seniority was not found to be a major factor impacting HCWs’ knowledge level or HAIs [51]. Another study found longer working experience to be negatively and independently correlated with knowledge and HAIs [52]. The reason for the increased risk is partially attributed to the frequency and riskiness of the performed procedures and the increase in career seniority [52]. Further research is required to fully understand the nature of the association between seniority and HAIs due to the inconsistent results in the literature.

Although our study yielded valuable insights, there are limitations. First, response bias is an evident weakness in these findings. Participants may be motivated and interested in the subject, or they may have strong feelings about it. This would likely impact their self-reported responses. Second, Even though a review of the literature was conducted before the study began to determine risk variables for HAIs, confounding factors that are unknown or were overlooked likely exist. Third, this study used a random sample from several hospitals in the Riyadh region. Finally, although random sampling can improve the study’s representativeness and reduce bias, a larger sample size from various regions is preferred to make it more generalizable.

## Conclusion

HAIs pose a serious and multifaceted threat to the global healthcare system. The findings indicated that HCWs who have poor knowledge of IPC, who reported no previous IPC applied training, and who have more years of clinical work experience have a greater risk of HAIs. Identifying the risk factors of HAIs would decrease hospital stay durations, lower mortality rates, reduce costs for drugs and supplies, and lessen the disease burden for patients, especially in sepsis situations. Thus, legislators and representatives of the healthcare field should prioritize the prevention of infections linked to healthcare, paying particular attention to tailored and applied IPC initiatives. Furthermore, IPC would be more clearly established within the context of patient and workplace safety if promoted inside an organization and HCWs at all levels were involved in IPC "ownership."

## Supporting information

S1 Dataset (XLSX)
